# Additional Comments on “Testing a Culturally Adapted Colorectal Cancer Screening Decision Aid Among American Indians: Results from a Pre–Post Trial”

**DOI:** 10.1089/heq.2020.0035

**Published:** 2021-05-25

**Authors:** Kazuhiko Kotani, Adam Lebowitz

**Affiliations:** ^1^ Division of Community and Family Medicine, Center for Community Medicine, Jichi Medical University, Shimotsuke-City, Japan.; ^2^ General Studies Department, Jichi Medical University, Shimotsuke-City, Japan.

To the Editor:

We read the study by Frerichs et al.^1^ concerning a culturally sensitive colorectal cancer (CRC) screening decision aid campaign targeting American Indians (Native Americans) with great interest in light of the increase in CRC-related deaths worldwide.^2^ Culture is basically associated with environmental and lifestyle factors as well as health care access, all of which are related to the incidence of CRC.^2^ Therefore, adapting methods in consideration of a given population's culture—especially important for a population with close community networks—in the health care system will offer new insights into development, efficacy, and delivery of the CRC screening instruments themselves.

Interestingly, their report showed that American Indians preferred colonoscopy (60%) to the fecal occult blood test (FOBT; 21%).^1^ However, besides showing that more education leads to more knowledge and positive attitude, their report still leaves much unclear. What precisely in the intervention is responsible for these results? The authors noted that collectivism and privacy were main concerns of this community, and “concepts were explained using easy-to-understand media” from community members.^1^ Wouldn't an approach incorporating these components be positive for other communities? For example, in Japan, the FOBT-first screening system has been widely accepted, suggesting the preference of the general population for the FOBT despite risk of false-positive results.^3^ According to a previous study of other ethnic populations, the rates of choosing FOBT or colonoscopy were almost equal (35% for FOBT and 41% for colonoscopy) and subpopulations who were not interested in screening tests tended to choose FOBT.^4^

Other factors may be associated with these disparate results. That is, preferences concerning screening tests might partly stem from the system design. Is screening simply following national mandates, or is community consensus considered? How are risks such as intestinal bleeding and perforation^5^ explained? What is the role of health care providers and insurance providers? This last point seems important since cost influences actual implementation of testing—almost 75% of Frerichs et al.'s sample had yearly income <USD 40,000,^1^ so colonoscopy's high cost might deter some individuals in practice despite stated preference.

Overall, the authors are to be commended for creating a culturally sensitive awareness campaign about CRC as an advanced view of screening system. We look forward to further in-depth research (considering the concerns presented here) linking better community health outcomes with this decision aid, which also clarifies effective mechanisms in the intervention. This may reduce the inequality in CRC-related deaths.

## Abbreviations Used

CRC: colorectal cancer
FOBT: fecal occult blood test
